# Follow-Up Study of the Chest CT Characteristics of COVID-19 Survivors Seven Months After Recovery

**DOI:** 10.3389/fmed.2021.636298

**Published:** 2021-03-01

**Authors:** Mengqi Liu, Fajin Lv, Yang Huang, Kaihu Xiao

**Affiliations:** ^1^Department of Radiology, The First Affiliated Hospital of Chongqing Medical University, Chongqing, China; ^2^Department of Cardiology, Chongqing University Three Gorges Hospital, Chongqing, China

**Keywords:** coronavirus disease 2019, computed tomography, follow-up, pulmonary fibrosis, cardiopulmonary exercise testing

## Abstract

**Background:** It has remained a concern whether any long-term pulmonary sequelae exist for COVID-19 survivors.

**Methods:** Forty-one patients (22 men and 19 women, 50 ± 14 years) confirmed with COVID-19 performed follow-up chest CT and cardiopulmonary exercise testing at 7 months after discharge. Patients were divided into fibrosis group and non-fibrosis group according to the evidence of fibrosis on follow-up CT. The clinical data and the CT findings were recorded and analyzed.

**Results:** The predominant CT patterns of abnormalities observed at 7 months after discharge were parenchymal band (41%), interlobular septal thickening (32%), and traction bronchiectasis (29%). Sixty-one percent of the patients achieved complete radiological resolution, and 29% of patients developed pulmonary fibrosis. Compared with the patients in the non-fibrosis group, the patients in the fibrosis group were older, with a longer hospital stay, a higher rate of steroid and mechanical ventilation therapy, lower levels of lymphocyte and T cell count, higher levels of D-dimer and lactic dehydrogenase, and higher quantitative CT parameters (opacity score, volume of opacity, and percentage of opacity) at discharge. Besides, oxygen consumption and metabolic equations were decreased and ventilatory equivalent for carbon dioxide was increased in patients in the fibrosis group. Logistic regression analyses revealed that age, steroid therapy, presence of traction bronchiectasis on chest CT at discharge, and opacity score at discharge, were independent risk factors for developing pulmonary fibrosis at 7 months after discharge. Receiver operating characteristic analysis revealed that the combined clinical-radiological model was better than the clinical-only model in the prediction of pulmonary fibrosis.

**Conclusions:** The chest CT lesions could be absorbed without any sequelae for most patients with COVID-19, whereas older patients with severe conditions are more prone to develop fibrosis, which may further lead to cardiopulmonary insufficiency. The combined clinical-radiological model may predict the formation of pulmonary fibrosis early.

## Introduction

Since late December 2019, coronavirus disease 2019 (COVID-19) has emerged and promptly spread throughout the world. As of 1 February 2021, there have been over 102.3 million confirmed cases and 2.2 million deaths reported globally since the start of the pandemic (1). Thin-section chest CT scans have been making a significant contribution to the disease assessment. Currently, the radiological characteristics of COVID-19 have been extensively studied. The typical chest CT features of COVID-19 include ground glass opacities (GGO), consolidation, and interlobular septal thickening with peripheral distribution (2–4). To date, many patients have been recovered and discharged. It has remained a concern to the public whether any pulmonary sequelae exist for COVID-19 survivors. Wang and colleagues reported that clinical sequelae of patients on 3-month follow-up were common, including respiratory symptoms, cardiovascular-related symptoms, and psychosocial symptoms (5). Besides, recent studies have shown that pulmonary fibrosis may develop in patients with COVID-19 short term after discharge (6–8). However, the number of discharged COVID-19 patients keeps increasing worldwide but the definite long-term radiological outcomes of the patients after discharge are scarcely described in the literature. Herein, we present the results of the 7-month follow-up chest CT in patients discharged with COVID-19.

## Methods

### Patients

This is a prospective observational study performed in patients discharged with COVID-19 from Chongqing University Three Gorges Hospital between February 10, 2020 and March 23, 2020. This study was approved by the local institutional review board. Written informed consents were obtained from all the patients enrolled in the study. The diagnostic and discharge criteria of COVID-19 pneumonia followed the latest guideline of Diagnosis and Treatment Program of COVID-19 released by the National Health Commission of the People's Republic of China (9). Clinical data of the patients were reviewed.

### Image Acquisition and Radiological Follow-Up

All the patients were imaged with a multi-detector CT scanner (uCT 510, United Imaging, China) using the following parameters: 120 kVp, 150 mA, 1.5 mm collimation, reconstruction matrix of 512 × 512, and slice thickness of 1.0 mm. All the patients were scanned in a supine position with a single inspiratory phase. The scanning range included the whole chest from the first ribs to the diaphragm. All the patients were examined without injection of contrast media. Images were obtained with mediastinal (width, 400 HU; level, 30 HU) and parenchymal (width, 1600 HU; level, −600 HU) window settings. We reviewed three chest CT scans for each patient: the CT examination at discharge, the CT examination at 3 months after discharge, and the CT examination at 7 months after discharge.

### Image Interpretation

**Figure 1:**
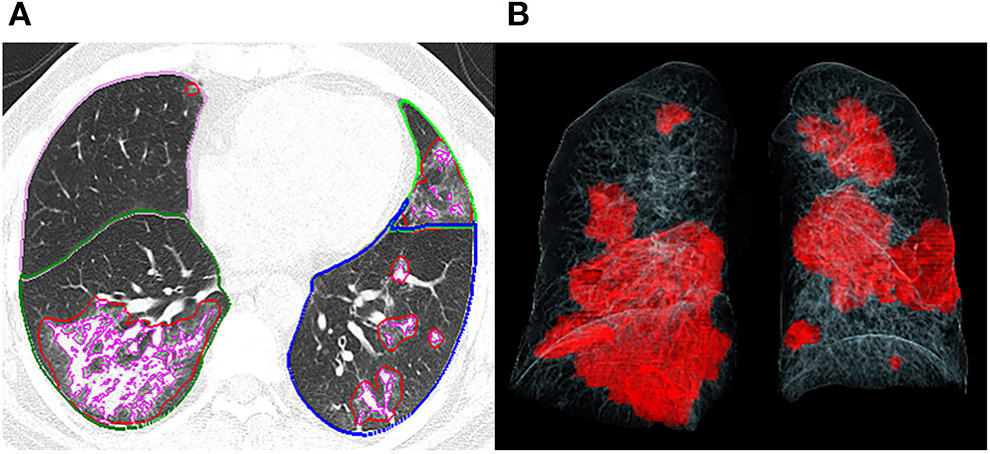
Representative postprocessing result of chest CT scan performed with Siemens CT Pneumonia Analysis. A 56-year-old male with COVID-19 (Opacity score = 5, volume of opacity = 671.6 ml, percentage of opacity = 15.1%). **(A)** Axis plane of chest CT shows bilateral ground glass opacities. **(B)** 3D-visualization of CT volume rendering technique shows the diffuse extent of opacities.

Three thoracic radiologists (with 8, 12, and 25 years of experience, respectively) blinded to the clinical data reviewed the CT images independently, and the discrepancies were resolved by discussion and consensus. The CT images of each patient were assessed and compared for the presence of the following features: GGO, consolidation, crazy paving pattern, air bronchogram, nodules, interlobular septal thickening, irregular interfaces, reticular pattern, parenchymal bands, and traction bronchiectasis. All the above glossaries were defined according to the Fleischner Society (10). In addition, an artificial intelligence software (CT Pneumonia Analysis, Siemens Healthineers, Siemens, Erlangen, Germany) was employed to automatically identify and quantify hyperdense areas of the lung (Figure 1), and the following quantitative parameters were calculated for each CT scan: opacity score, volume of opacity, and percentage of opacity (the ratio of volume of opacity to lung volume). Each of the 5 lung lobes was scored automatically 0–4 as 0 (≤1% involvement), 1 (2–25%), 2 (26–50%), 3 (51–75%), and 4 (>75%). The total opacity score was the sum of the five lobar scores and ranged from 0 to 20. Patients were divided into two groups according to the evidence of fibrosis on the 7-month follow-up CT imaging: fibrosis group and non-fibrosis group. Fibrosis on chest CT was defined as a combination of findings including parenchymal bands, reticular pattern, and traction bronchiectasis (8, 11, 12). Clinical and radiological data between the two groups were subsequently compared.

### Cardiopulmonary Exercise Testing

Cardiopulmonary exercise testing was performed at the latest follow-up. Forced expiratory volume in 1 s (FEV1) and forced vital capacity (FVC) were measured using a turbine spirometer (Cosmed, Quark CPET, Rome, Italy). The incremental exercise was done on a treadmill (Cosmed, Quark CPET, Rome, Italy). Patients were encouraged to exercise until symptoms were intolerable. Peak oxygen uptake (VO_2_), minute ventilation (VE), and carbon dioxide production (VCO_2_) were measured using a Cosmed Face Mask (Quark CPET, Rome, Italy). The anaerobic threshold (AT) was defined as the VO_2_ level where the VE/VCO2 decreased or remained constant while the ventilatory equivalent to oxygen (VE/VO_2_) persistently increased. Finally, the following parameters were recorded for each object: FEV1/FVC, Peak VO_2_ (mL/min/kg), VO_2_ at AT (mL/min/kg), peak metabolic equations (METs), and VE/VCO_2_ slope.

### Statistical Analysis

Statistical analyses were performed with the SPSS for Windows software package (version 17.0, SPSS Inc) and MedCalc statistical software (Med-Cale Software, Mariakerke, Belgium). The normality of the distribution was checked using a Kolmogorov-Smirnov test. Continuous variables were expressed as mean ± standard deviation (SD) or median [interquartile range (IQR)] and compared with independent *t*-test or Mann-Whitney U test. Categorical variables were expressed as absolute and relative frequencies (%) and compared by the χ^2^ test or Fisher's exact test between groups. Spearman's correlations were performed to evaluate the relationship between the three quantitative CT parameters (opacity score, volume of opacity, and percentage of opacity) and the laboratory results. Logistic regression analyses were performed to identify independent risk factors for developing pulmonary fibrosis at 7 months after discharge. Besides, receiver operating characteristic curves (ROCs) were constructed for the independent risk factors for predicting pulmonary fibrosis. Optimum cut-off point, sensitivity, specificity, and area under the curve (AUC) of each indicator were calculated. Then, comparisons of ROC curves between clinical characteristics alone and combined clinical-CT characteristics were performed using the non-parametric approach of DeLong et al. A *P*-value <0.05 was considered statistically significant.

## Results

### Clinical Characteristics of Patients

A total of 41 COVID-19 survivors (male: 22, female: 19) were enrolled in the present study, including 26 patients of moderate type and 15 patients of severe type (including 13 severe diseases and 2 critical diseases). Of these patients, 12 patients (29%, male: 7, female: 5) with evidence of fibrosis on the 7-month follow-up CT were designated as the fibrosis group, and the remaining 29 patients (71%, male: 15, female: 14) without evidence of fibrosis as the non-fibrosis group. The average age was 50 years old. The average days from symptom onset to admission were 6 days. The most prevalent initial symptoms were fever (66%) and cough (56%). Less common symptoms were sputum, dyspnea, headache, dyspnea, and muscle ache. Hypertension (7%) and chronic obstructive pulmonary disease (5%) were the most common concomitant diseases. Steroid hormone was given to 7 (17%) patients at a median of 5 days (IQR 4–10 days, prednisolone 40 mg b.i.d.), while 7 patients received mechanical ventilation at a median of 7 days (IQR 3–7 days). The patients were ventilated with continuous positive airway pressure (CPAP) via facemask at 10–12 cm H_2_0 at a fraction of inspired oxygen (FIO2) of 40–60%. The average hospital stay was 18 days. Subgroup analysis revealed that patients in the fibrosis group were older (63 ± 12 years) than those in the non-fibrosis group (45 ± 11 years) (*p* < 0.001). Besides, patients with severe type were more likely to develop pulmonary fibrosis at 7 months follow-up (*p* = 0.010). There were no obvious differences in the clinical symptoms and the proportion of patients with comorbidities between the two groups. However, compared with the non-fibrosis group, the fibrosis group had lower levels of lymphocyte count (*p* = 0.019) and T cell count (*p* = 0.022) at discharge, and had higher levels of D-dimer (*p* < 0.001) and lactic dehydrogenase (LDH, *p* = 0.037). In addition, more patients in the fibrosis group were mechanically ventilated (*p* < 0.001), and more patients were treated with steroids (*p* < 0.001). The details are summarized in Table 1.

**Table 1:** Clinical and laboratory characteristics of the patients included in the present study.

**Characteristics**	**Total (*N* = 41)**	**Fibrosis group (*N* = 12)**	**Non-fibrosis group (*N* = 29)**	***P*-value**
**Sex**				
Male	22 (54%)	7 (58%)	15 (52%)	0.259
Female	19 (46%)	5 (42%)	14 (48%)	
Age (years)	50 ± 14	63 ± 12	45 ± 11	**<0.001**
**Severity**				
Moderate	26 (63%)	4 (33%)	22 (76%)	**0.010**
Severe	15 (37%)	8 (67%)	7 (24%)	**0.010**
**Comorbidity**				
Diabetes	1 (2%)	1 (8%)	0 (0%)	0.116
Hypertension	3 (7%)	2 (17%)	1 (3%)	0.139
Chronic obstructive pulmonary disease	2 (5%)	1 (8%)	1 (3%)	0.509
Cardiovascular disease	1 (2%)	1 (8%)	0 (0%)	0.116
Hepatic disease	1 (2%)	0 (0%)	1 (3%)	0.505
≥1 comobidity	7 (17%)	4 (33%)	3 (7%)	0.075
**Symptoms**				
Fever	27 (66%)	8 (67%)	19 (66%)	0.944
Cough	23 (56%)	6 (50%)	17 (59%)	0.613
Sputum	9 (22%)	2 (17%)	7 (24%)	0.599
Fatigue	10 (24%)	5 (42%)	5 (17%)	0.127
Dyspnea	5 (12%)	3 (25%)	2 (7%)	0.107
Headache	10 (24%)	3 (25%)	7 (24%)	0.953
Muscle ache	12 (29%)	3 (25%)	9 (31%)	0.699
**Lab test at discharge**				
White blood cell count (3.5–9.5 × 10^9^/L)	5.59 ± 1.95	5.58 ± 2.26	5.59 ± 1.85	0.988
Lymphocyte count (1.1–3.2 × 10^9^/L)	1.22 ± 0.49	0.97 ± 0.43	1.32 ± 0.48	**0.019**
T cell count (699–2,540 × 10^6^/L)	784.57 ± 248.75	635.00 ± 190.61	844.40 ± 246.95	**0.022**
B cell count (90–660 × 10^6^/L)	112.74 ± 60.14	89.60 ± 43.34	122.00 ± 64.09	0.153
NK cell count (90–590 × 10^6^/L)	135.09 ± 116.39	91.51 ± 57.53	152.52 ± 129.77	0.164
D-dimer (0–0.55mg/L)	0.41 (0.22–0.89)	1.02 (0.42–2.96)	0.31 (0.20–0.72)	**<0.001**
Lactic dehydrogenase (120–250 IU/L)	192.96 ± 56.42	220.25 ± 80.41	182.46 ± 41.42	**0.037**
C-reactive protein (0–11 mg/L)	3.21 (1.83–8.79)	3.91 (2.28–13.68)	3.08 (1.45–7.68)	0.523
Procalciton (<0.046 ng/mL)	0.031 (0.025–0.050)	0.030 (0.020–0.069)	0.032 (0.025–0.043)	0.883
Interleukin-6 (0–7 pg/mL)	4.97 (0.00–22.26)	13.81 (0.00–46.45)	3.13 (0.00–9.79)	0.383
**Treatment**				
Steroid	7 (17%)	6 (50%)	1 (3%)	**<0.001**
Mechanical ventilation	7 (17%)	4 (33%)	3 (7%)	**<0.001**
Days from onset to admission	6 ± 4	6 ± 3	6 ± 4	0.947
Days from discharge to last follow-up	219 ± 11	213 ± 11	220 ± 10	0.066
Hospital stay (day)	18 ± 7	23 ± 8	17 ± 6	**0.008**

*Data are expressed as mean ± standard deviation, median (interquartile range), and n (%). Bold values indicate p < 0.05*.

### Chest CT Evaluation

**Figure 2:**
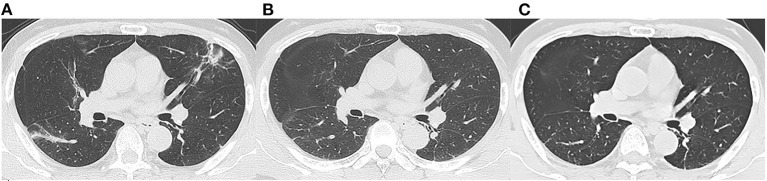
Follow-up chest CT images of a 52-year-old male with COVID-19. **(A)** At discharge, CT imaging shows parenchymal bands, irregular interfaces, and traction bronchiectasis in left upper lobe and right lower lobe. The lesions are almost revolved on the 3-month follow-up **(B)** and 7-month follow-up **(C)** CT. This patient was enrolled in non-fibrosis group.

**Figure 3:**
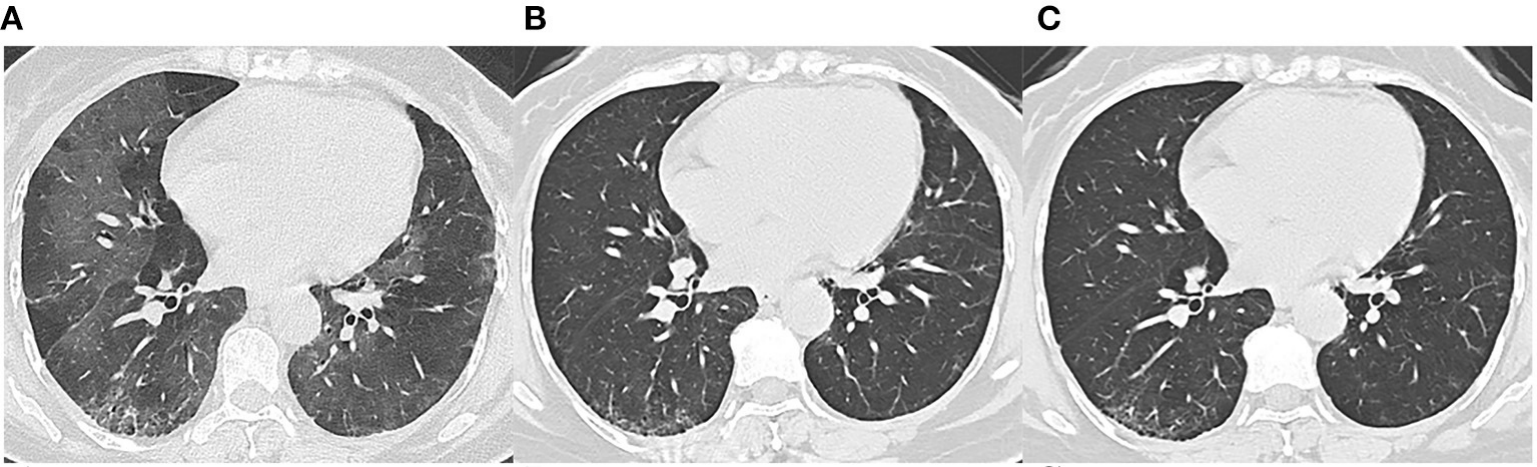
Follow-up chest CT images of a 48-year-old female with COVID-19. **(A)** At discharge, CT imaging shows crazy paving pattern, parenchymal bands, irregular interfaces, and reticular pattern in bilateral lungs. **(B)** 3-month follow-up CT shows parenchymal bands, irregular interface, and traction bronchiectasis in the right lower lobe, which indicates fibrosis. **(C)** The lesions on 7-month follow-up CT are still present. This patient was enrolled in fibrosis group.

We reviewed three CT scans for each patient. The average interval between discharge and latest follow-up was 219 days, and the average intervals of the two subgroups did not differ statistically (*p* = 0.066). The dynamic changes of chest CT features were listed in Table 2. As shown in Table 2, the predominant chest CT features observed at discharge included parenchymal band (73%), GGO (71%), interlobular septal thickening (68%), consolidation (46%), and irregular interface (46%). The less common CT features included reticular pattern (39%) and traction bronchiectasis (29%). The rare CT features were air bronchogram (7%) and crazy paving pattern (5%). Quantitative CT parameters calculated by the artificial intelligence software revealed that the median opacity score was 4.0 (IQR 2.0–5.0), the median volume of opacity was 178.0 ml (IQR 41.4–422.0 ml), and the median percentage of opacity was 4.3% (IQR 1.0%−12.4%). After discharge, all the quantitative CT parameters gradually decreased with time. The median opacity score, volume of opacity, and percentage of opacity on the latest follow-up CT (7 months after discharge) were decreased to 0.0 (IQR 0.0–1.0), 0.9 (IQR 0.0–12.8) ml, and 0.0% (IQR 0.0–0.3%), respectively. Twenty-five patients (61%) achieved complete radiological resolution on the 7-month follow-up CT. GGO (12%) and consolidation (10%) were almost resolved on the 7-month follow-up CT, while evidence of fibrosis, such as parenchymal band (41%), interlobular septal thickening (32%), reticular pattern (12%), and traction bronchiectasis (29%), were still obvious (Figures 2, 3).

**Table 2:** Chest CT findings in the patients with COVID-19.

	**At discharge**				**3 months after discharge**			**7 months after discharge**		
	**Total (*N* = 41)**	**Fibrosis group (*N* = 12)**	**Non-fibrosis group (*N* = 29)**	***P*-value**	**Total (*N* = 41)**	**Fibrosis group (*N* = 12)**	**Non-fibrosis group (*N* = 29)**	**Total (*N* = 41)**	**Fibrosis group (*N* = 12)**	**Non-fibrosis group (*N* = 29)**
Opacity score	4.0 (2.0–5.0)	6.7 (5.0–9.0)	2.9 (1.0–4.8)	**0.002**	0.0 (0.0–2.0)	2.0 (0.0–3.0)	0.4 (0.0–0.8)	0.0 (0.0–1.0)	1.7 (0.5–3.0)	0.2 (0.0–0.3)
Volume of opacity (ml)	178.0 (41.4–422.0)	602.0 (156.0–1080.0)	183.0 (6.0–249.0)	**0.009**	1.4 (0.0–19.0)	51.0 (1.0–67.0)	20.0 (0.0–33.0)	0.9 (0.0–12.8)	33.0 (3.0–41.0)	5.0 (0.0–18.0)
Percentage of opacity	4.3 (1.0–12.4)	19.4 (3.5–32.5)	5.3 (0.1–8.8)	**0.007**	0.0 (0.0–0.5)	1.7 (0.0–1.9)	0.7 (0.0–0.8)	0.0 (0.0–0.3)	1.1 (0.0–1.6)	0.2 (0–1.0)
GGO	29 (71%)	11 (92%)	18 (62%)	0.058	11 (27%)	7 (58%)	4 (14%)	5 (12%)	4 (33%)	1 (3%)
Consolidation	19 (46%)	8 (67%)	11 (38%)	0.093	6 (15%)	4 (33%)	2 (7%)	4 (10%)	3 (25%)	1 (3%)
Crazy paving pattern	2 (5%)	1 (8%)	1 (3%)	0.509	0 (0%)	0 (0%)	0 (0%)	0 (0%)	0 (0%)	0 (0%)
Air bronchogram	3 (7%)	2 (17%)	1 (3%)	0.139	0 (0%)	0 (0%)	0 (0%)	0 (0%)	0 (0%)	0 (0%)
Interlobular septal thickening	28 (68%)	12 (100%)	16 (55%)	**0.005**	17 (41%)	12 (100%)	5 (17%)	13 (32%)	12 (100%)	1 (3%)
Irregular interface	19 (46%)	9 (75%)	10 (34%)	**0.018**	6 (15%)	6 (50%)	0 (0%)	5 (12%)	5 (42%)	0 (0%)
Reticular pattern	16 (39%)	12 (100%)	4 (14%)	**<0.001**	10 (24%)	10 (83%)	0 (0%)	5 (12%)	5 (42%)	0 (0%)
Parenchymal band	30 (73%)	12 (100%)	18 (62%)	**0.013**	22 (54%)	12 (100%)	10 (34%)	17 (41%)	12 (100%)	5 (17%)
Traction bronchiectasis	12 (29%)	10 (83%)	2 (7%)	**<0.001**	14 (34%)	12 (100%)	2 (7%)	12 (29%)	12 (100%)	0 (0%)

*Data are median (interquartile range) or n (%). Bold values indicate p < 0.05*.

The imaging features on chest CT at discharge were further compared between the fibrosis group and the non-fibrosis group (Table 2). Compared with the non-fibrosis group, the fibrosis group had higher levels of opacity score (*p* = 0.002), volume of opacity (*p* = 0.009), and percentage of opacity (*p* = 0.007). Moreover, more patients in the fibrosis group manifested interlobular septal thickening (*p* = 0.005), irregular interface (*p* = 0.018), reticular pattern (*p* < 0.001), parenchymal band (*p* = 0.013), and traction bronchiectasis (*p* < 0.001).

### Correlations of CT Quantitative Parameters With Laboratory Tests

Spearman's correlations were performed to explore the relationship between the three quantitative CT parameters and the laboratory results at discharge. As shown in Table 3, all three quantitative CT parameters were positively correlated with white blood cell count, C-reactive protein, D-dimer, and LDH.

**Table 3:** Correlations of CT quantitative parameters with laboratory tests.

		**WBC**	**CRP**	**PCT**	**DD**	**LDH**	**Lymphocyte**	**T cell**
Opacity score	Correlation coefficient r	0.401	0.337	−0.195	0.343	0.720	−0.214	−0.254
	*p*-value	0.014	0.042	0.248	0.035	0.000	0.197	0.124
Volume of opacity	Correlation coefficient r	0.401	0.397	−0.184	0.344	0.604	−0.079	−0.114
	*p*-value	0.014	0.015	0.276	0.035	0.000	0.636	0.495
Percentage of opacity	Correlation coefficient r	0.464	0.409	−0.104	0.348	0.722	−0.181	−0.218
	*p*-value	0.004	0.012	0.540	0.032	0.000	0.276	0.188

*WBC: white blood cell; CRP: C-reactive protein; PCT: procalciton; DD: D-dimer; LDH: lactic dehydrogenase*.

### Logistic Regression and ROC Curve Analysis

**Figure 4:**
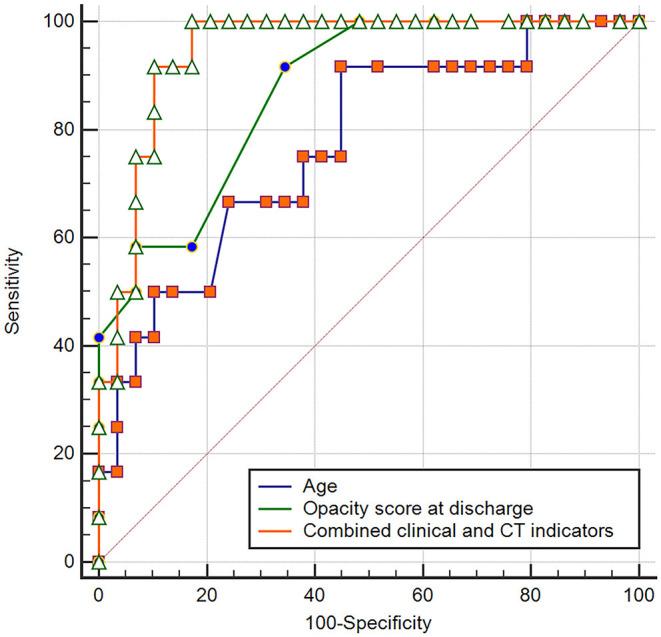
Receiver operating curve for pulmonary fibrosis prediction in patients with COVID-19 using age or opacity score at discharge alone and combined clinical-CT indicators.

Logistic regression analyses revealed that age (odds ratio = 1.078, *p* = 0.049), steroid therapy (odds ratio = 12.880, *p* = 0.010), opacity score at discharge (odds ratio = 1.565, *p* = 0.034), and presence of traction bronchiectasis at discharge (odds ratio = 13.570, *p* = 0.012), were independent risk factors for developing pulmonary fibrosis at 7 months after discharge, while other clinical or CT indicators (sex, disease severity, lymphocyte count, T cell count, D-dimer, LDH, hospital stay, volume of opacity, percentage of opacity, parenchymal band, reticular pattern, ventilation therapy, days of ventilation) were not independent risk factors associated with fibrosis. For the prediction of pulmonary fibrosis at 7 months after discharge, ROC analysis yielded AUC values of 0.770, 0.782, 0.848, and 0.871 for age (cutoff point: >48 years), steroid therapy, traction bronchiectasis at discharge, and opacity score at discharge (cutoff point: >4), respectively (Table 4). When combined the above clinical and CT indicators, the AUC value of the combined model was increased to 0.945 (Figure 4), which was significantly different from that of age (*p* = 0.031), but not significantly different from that of opacity score (*p* = 0.109).

**Table 4:** ROC analysis results for independent variables for predicting pulmonary fibrosis at 7 months after discharge.

**Variable**	**AUC (upper and lower limit)**	**SE**	**Se**	**Sp**	**Optimal cut point**
Age	0.770 (0.612–0.887)	0.0828	91.67	55.17	>48
Steroid therapy	0.782 (0.625–0.895)	0.0767	66.67	89.66	
Traction bronchiectasis	0.848 (0.701–0.941)	0.0649	83.33	86.21	
Opacity score at discharge	0.871 (0.729–0.955)	0.0549	91.67	65.52	>4
Combined clinical and CT	0.945 (0.826–0.992)	0.0329	91.67	82.76	

*AUC: area under curve; SE: Standard error; Se: sensitivity; Sp: specificity*.

### Cardiopulmonary Exercise Testing

There was no significant difference in FEV1/FVC and VO_2_ AT between the fibrosis group and the non-fibrosis group. However, compare with the non-fibrosis group, the fibrosis group had decreased VO_2_/kg and METs and increased VE/VCO_2_. The details are summarized in Table 5.

**Table 5:** Results of cardiopulmonary exercise testing on patients with COVID-19.

	**Fibrosis group**	**Non-fibrosis group**	***P*-value**
FEV1/FVC	98.0 ± 6.8	99.0 ± 8.1	0.741
VO2/Kg	16.4 ± 3.6	20.2 ± 3.7	0.009
VO2 AT	14.6 ± 3.7	16.0 ± 3.5	0.317
METs	4.7 ± 1.0	5.8 ± 1.0	0.010
VE/VCO2	30.6 ± 4.0	26.3 ± 3.2	0.003

*Data are presented as mean ± standard deviation*.

*FEV1, forced expiratory volume in 1 second; FVC, forced vital capacity; VO_2_, oxygen consumption; AT, anaerobic threshold; METs, metabolic equations; VE/VCO_2_, ventilatory equivalent for carbon dioxide*.

## Discussion

After severe acute respiratory syndrome (SARS) outbreak in 2003, plenty of patients recovered. However, radiological abnormalities were detected in more than 70% of patients who recovered from SARS at 4–6 months after admission to the hospital (13, 14), and long-term pulmonary sequelae were also reported in previous studies (15, 16). So this may raise an important question for doctors: are there any long-term pulmonary sequelae in patients recovering from COVID-19? Up to now, few reports have described the sequelae of COVID-19 survivors (5–7, 17–20), and the long-term radiological changes have not been well-studied. In our study, we presented the results of 7-month follow-up chest CT in patients with COVID-19, and we compared clinical data and chest CT between patients with or without pulmonary fibrosis.

Clinically, patients in the fibrosis group were older than those in the non-fibrosis group, similar to SARS (11), which was in line with a previous study (8). Besides, the fibrosis group had a longer time of hospital stay. Moreover, more patients in the fibrosis group were severe type and treated with steroids and ventilator, and the fibrosis group had lower levels of lymphocyte count and T cell count and higher levels of D-dimer and LDH at discharge, which indicated that the patients with fibrosis may have more severe conditions with COVID-19. Furthermore, we found all the three quantitative CT parameters were positively correlated with white blood cell count, C-reactive protein, D-dimer, and LDH, which demonstrated that these quantitative CT parameters were reliable indicators of disease severity.

In our study, the chest CT abnormalities were still apparent at discharge. However, the pulmonary lesions had been gradually absorbed after discharge. Compared with the abnormalities found on the CT scans at discharge, the cases with GGO, consolidation, and air bronchogram were reduced from 71, 46, and 7%, to 12, 10, and 0% of cases on the 7-month follow up CT, respectively. Correspondingly, the median opacity score, volume of opacity, and percentage of opacity, were reduced from 4.0, 178 ml, and 4.3%, to 0.0, 0.9 ml, and 0.0%, respectively. Up to 66% of patients (86% of patients in the non-fibrosis group) achieved complete radiological resolution. Similarly, it was reported that the pulmonary lesions of 53.0% of COVID-19 patients would be fully absorbed at 3 weeks after discharge (18). Our results demonstrated that pulmonary lesions in the majority of COVID-19 patients could be reversible without any sequelae.

However, the CT manifestations of pulmonary fibrosis (interlobular septal thickening, irregular interface, parenchymal band, and traction bronchiectasis) were still apparent on the 7-month follow-up CT. As reported, pulmonary fibrosis may develop in the early stage in discharged patients with SARS (11), and the fibrosis may be long-persistent (15), whereas the pulmonary fibrosis in discharged COVID-19 patients may be absorbed with time (6), which was consistent with our study (Figure 2). The reversibility of fibrosis probably indicated that the pulmonary fibrosis on chest CT did not signify actual pathologic fibrosis (14), and thus whether these lesions would completely disappear required further observation.

In our cohort, logistic regression analyses revealed that age, steroid therapy, presence of traction bronchiectasis, and opacity score at discharge, were independent risk factors for developing pulmonary fibrosis on 7-month follow-up CT. We could speculate that the above four indicators might be early predictors of pulmonary fibrosis in patients recovered from COVID-19. Further ROC analysis revealed that the combined clinical-radiological model was better than the clinical-only model in the prediction of pulmonary fibrosis. Our result should be validated by further large-scale studies.

Zhao and colleagues reported that lung function abnormalities were detected in 25% of COVID-19 patients at 3 months after discharge (21). Cardiopulmonary exercise testing provides integrated data about cardiovascular, ventilatory and gas exchange, metabolic, and skeletal muscle response to the physical effort (22). So cardiopulmonary exercise testing can provide more physiological information than lung function tests. We found VO_2_/kg and METs were decreased and VE/VCO_2_ was increased in patients in the fibrosis group, which may imply cardiopulmonary insufficiency resulting from fibrosis.

Our study had several limitations. Firstly, the sample size was quite small. However, our further studies will consider increasing the sample size of discharged patients on the 1-year follow-up. Secondly, pulmonary fibrosis had not been confirmed by pathology even though the CT manifestations were typical. We will continue to follow up these patients to confirm whether the radiological fibrosis could be further absorbed. Finally, we included very few patients with critically severe type, which may underestimate the incidence rate of pulmonary fibrosis. We will try to enroll more patients with severe type in future studies.

In conclusion, the most common imaging patterns of COVID-19 pneumonia at 7 months after discharge are interlobular septal thickening, parenchymal band, traction bronchiectasis, and reticular pattern. The chest CT lesions could be absorbed without any sequelae for most patients with COVID-19, whereas older patients with severe conditions are more prone to develop fibrosis, which may further lead to cardiopulmonary insufficiency. The quantitative CT parameters (opacity score, volume of opacity, and percentage of opacity) are reliable indicators of disease severity. Age >48 years old, steroid therapy, presence of traction bronchiectasis on chest CT at discharge, and opacity score at discharge >4, are the independent risk factors associated with fibrosis. The combined clinical-radiological model may be better than the clinical-only model in the prediction of developing pulmonary fibrosis on the 7-month follow-up.

## Data Availability Statement

The raw data supporting the conclusions of this article will be made available by the authors, without undue reservation.

## Ethics Statement

The studies involving human participants were reviewed and approved by The First Affiliated Hospital of Chongqing Medical University. The patients/participants provided their written informed consent to participate in this study.

## Author Contributions

ML and KX: conception and design. YH and KX: collection and assembly of data. ML, YH, and FL: analysis and interpretation of the data. ML: statistical expertise. ML and FL: drafting of the manuscript. ML and KX: critical revision of the article for important intellectual content. All authors had full access to all of the data in the study and take responsibility for the integrity of the data and the accuracy of the data analysis.

## Conflict of Interest

The authors declare that the research was conducted in the absence of any commercial or financial relationships that could be construed as a potential conflict of interest.
